# Assessing the relative and attributable risk of stressors to wetland condition across the conterminous United States

**DOI:** 10.1007/s10661-019-7313-7

**Published:** 2019-06-20

**Authors:** Alan T. Herlihy, Steven G. Paulsen, Mary E. Kentula, Teresa K. Magee, Amanda M. Nahlik, Gregg A. Lomnicky

**Affiliations:** 1 Department of Fisheries and Wildlife, Oregon State University, 104 Nash Hall, Corvallis, OR 97331 USA; 2 National Health and Environmental Effects Research Laboratory- Western Ecology Division, US Environmental Protection Agency, 200 SW 35th St, Corvallis, OR 97333 USA; 3 CSS Dynamac Corp., 200 SW 35th St, Corvallis, OR 97333 USA

**Keywords:** Wetlands, Ecological condition, Indicators of stress, Relative risk, Attributable risk, Stressor extent, Human disturbance, National Wetland Condition Assessment

## Abstract

We analyzed data from 967 randomly selected wetland sites across the conterminous United States (US) as part of the 2011 National Wetland Condition Assessment (NWCA) to investigate the relative and attributable risk of various stressors on wetland vegetation condition. Indicators of stress included six physical stressors (damming, ditching, filling/erosion, hardening, vegetation removal, and vegetation replacement) and two chemical stressors (soil phosphorus and heavy metals) that represent a wide range of human activities. Risk was evaluated nationally and within four aggregate ecoregions and four aggregate wetland types. Nationally, all of the stressors except soil heavy metals and phosphorus had a significant relative risk but values were always < 2 (a relative risk of two indicates that it’s twice as likely to have poor vegetation condition when the stressor is present relative to when it is absent). Among the different ecoregions or wetland types, no one stressor was consistently riskier; all of the stressors were associated with poor vegetation condition in one or another of the subpopulations. Overall, hardening had the highest attributable and relative risks in the most different subpopulations. Attributable risks above 25% were observed for vegetation removal in the Coastal Plain, hardening and ditching in the West, and hardening in Estuarine Woody wetlands. Relative risks above 3 were noted for heavy metals and soil phosphorus in the Interior Plains, and vegetation removal, vegetation replacement, and damming in Estuarine Woody wetlands. Relative and attributable risk were added to the data analyses tools used in the NWCA to improve the ability of survey results to assist managers and policy makers in setting priorities based on conditions observed on the ground. These analyses provide useful information to both individual site managers and regional-national policy makers.

## Introduction

The United States (US) Environmental Protection Agency (USEPA), in partnership with the States, is tasked with making assessments of all US surface waters. Clearly, a census of all surface waters in the US far exceeds monitoring resources available to individual states or the USEPA. In the past, most assessment of surface waters were made based on found data. Sample surveys using a probability design, however, are a cost-effective way of making statistically valid national-scale assessments (Larsen et al. 1994), and have been used with great effectiveness in a variety of fields to describe the characteristics of populations that are too large to census efficiently (e.g., political election polls). The use of sample survey approaches for characterizing water resources began in the late 1980s with the National Surface Water Survey to assess acidic deposition impacts (Landers et al. 1988; Kaufmann et al. 1991). USEPA conducted the first national-scale condition assessment of wetland resources in 2011 through implementation of the National Wetland Condition Assessment (NWCA, USEPA 2016a, USEPA 2016b). The NWCA uses a probability design and is one of the National Aquatic Resource Surveys (NARS) conducted by the USEPA to assess the nation’s waters. The NARS were designed to make quantitative estimates of the condition of surface waters throughout the US. To address this objective, a large number (~ 1000) of randomly selected lakes, streams, rivers, wetlands, or near coastal sites are visited each year during a defined index period. The NARS include streams and rivers, lakes, near coastal areas, and now wetlands (e.g., USEPA 2016c; USEPA 2016d).

One of the key objectives of the NWCA and all the NARS is to rank important human-caused or mediated stressors to aid in identification of policy or management priorities. Van Sickle et al. (2006) adapted a risk assessment approach to allow its use with survey data to quantify the relative risk from multiple indicators of stress (hereafter, stressors) for wadeable streams in the mid-Atlantic. They borrowed the relative risk terminology from medical epidemiology because most people are familiar with the concept as it relates to human health (e.g., a greater risk of developing heart disease if one has high cholesterol levels). Relative risk results are presented in terms of a relative risk ratio. For example, the relative risk for colorectal cancer is 2.24 if one first-degree relative had the disease and 3.97 if more than one first-degree relative had the disease (American Cancer Society 2017; Butterworth et al. 2006). In other words, if you have a strong family history of the disease, you are four times more likely to get it than a person with no family history. The relative risk values we have calculated for the NWCA can be interpreted as how much more likely a wetland is to have poor vegetation condition if a stressor level is high as opposed to not high. For example, a relative risk of 1 indicates no greater risk of poor condition regardless of stressor level, while a relative risk of 2 indicates that condition is twice as likely to be poor when a stressor level is high relative to when it is not. Applied to the NWCA, a relative risk analysis can be used to evaluate the relative effect of a wide variety of anthropogenic stressors on wetland condition. Relative risk analyses are also standard for reporting results in other EPA aquatic resource surveys (Paulsen et al. 2008; Van Sickle et al. 2006; Van Sickle and Paulsen 2008; Van Sickle 2013).

Attributable risk combines the concept of relative risk with the magnitude of the stressor extent. It provides an estimate of the proportion of the resource population in poor condition that might be reduced if high levels of a particular stressor were eliminated (Van Sickle and Paulsen 2008, Van Sickle 2013). The calculation of attributable risk makes three major assumptions involving causality (the stressor causes an increased probability of poor condition); reversibility (if the stressor is eliminated, causal effects will also be eliminated); and independence (stressors are independent of each other). A highly desirable feature of attributable risk is that it combines estimated stressor extent with relative risk into a single index to permit ranking the evaluated stressor indicators by the degree of their potential impact on the total resource.

In the 2011 NWCA, sites were sampled across the conterminous US to characterize wetland vegetation, soil chemistry, water chemistry, and presence of anthropogenic stressors. One of the primary goals of the NWCA was to evaluate the ecological condition of wetlands in the US and rank the anthropogenic stressors that might affect them. Data from surveys like the NWCA are an excellent tool that can be used to quantify stressor relative risk for an array of stressor indicators across large geographic areas because results can be extrapolated to the entire population of interest. In this paper, we use NWCA data to evaluate the relative and attributable risk from eight physical/chemical stressors on the ecological condition of a large, sampled wetland population distributed across the conterminous US and within distinct subpopulations of four wetland types and four ecoregions. Analysis of the NWCA data provides a unique opportunity to investigate relative and attributable risk in wetlands at large continental- and regional-scales using data collected for this purpose.

## Methods

### NWCA survey design

The purpose of the NWCA is to generate statistically valid and environmentally relevant reports on the condition of the Nation’s wetland resources every 5 years. The NWCA was designed to assess the regional ecological condition of broad groups or subpopulations of wetlands, rather than smaller spatial scales (e.g., individual states) or individual wetlands. The NWCA target population included wetlands of the conterminous US meeting the following criteria: tidal or nontidal wetted areas with rooted vegetation; water if present, less than 1 m deep; and the wetland not currently in crop production (Olsen et al. 2019).

Details of the NWCA survey design and site selection are described in the NWCA technical report (USEPA 2016a) and in Olsen et al. (2019). In brief, sample site selection was completed in two steps. A consistent national digital map of all wetlands in the conterminous US was not available; however, the US Fish & Wildlife Service conducts the National Wetland Status and Trends (S&T) survey periodically to assess wetland extent. The approximately 5000 4-mi^2^ plots from S&T were used to identify wetlands in the first step of site selection. In the second step, a generalized random tessellation stratified survey design (Stevens Jr. and Olsen 1999; Stevens Jr. and Olsen 2004) for an area resource was applied to the S&T wetland polygons and stratified by state with unequal probability of selection by NWCA wetland type (Olsen et al. 2019). The randomly selected sample sites from the NWCA survey design were screened using recent aerial photo interpretation and geographic information system analysis to eliminate locations not suitable for NWCA sampling (e.g., non-NWCA wetland types, non-wetlands, wetlands lost due to land cover change). During field reconnaissance, additional sites might be eliminated if, for example, they were a non-target type or could not be assessed due to accessibility or safety issues. Dropped sites were systematically replaced from a pool of replacement sites from the random design.

**Fig. 1:**
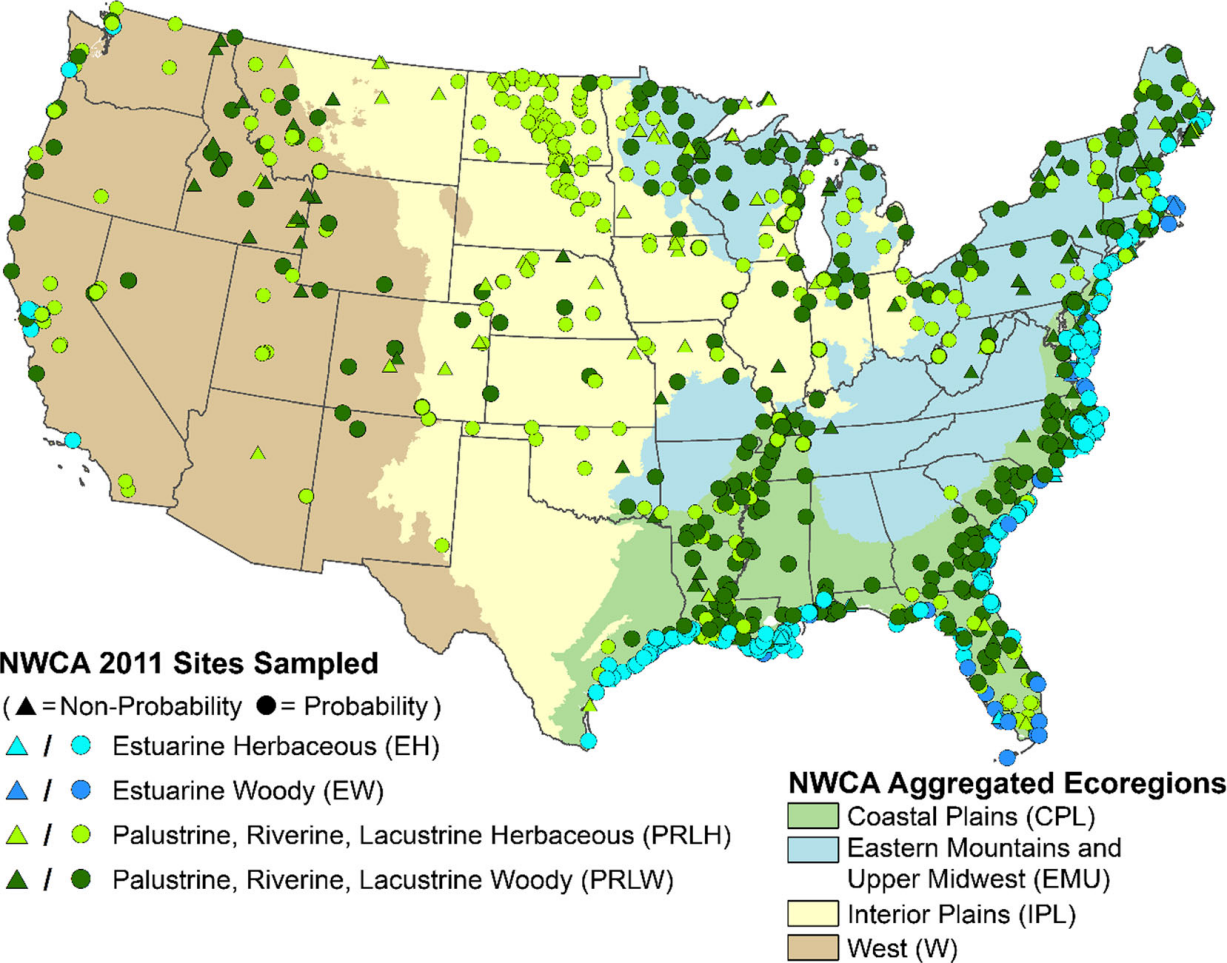
Location of sites sampled in the National Wetland Condition Assessment (NWCA) and the boundaries of the aggregated ecoregions used by the NWCA in the US

A total of 1138 sites were sampled in the NWCA, of which 967 (Table 1) were randomly selected probability sites used to make the national condition estimates in the NWCA report (USEPA 2016b). The other 171 sites were selected by other means for other objectives and were not used in our analysis of relative and attributable risk as they could not be used to infer national condition estimates (Herlihy et al. 2019a). The 967 probability sites used in our risk analysis were distributed throughout the conterminous US (Fig. 1). The spatial distribution across the country was not uniform but mirrored the national distribution of wetlands as represented in the S&T sample frame (Olsen et al. 2019).

**Table 1:** Number of sampled probability sites and estimated wetland area in the NWCA sampled population by NWCA aggregated ecoregions and aggregated wetland types

Full name	Code	Number of probability sites	Estimated wetland area (km^2^)
National	ALL	967	251,546
NWCA aggregated ecoregion			
Coastal Plain	CPL	513	125,025
Eastern Mountains and Upper Midwest	EMU	152	80,765
Interior Plains	IPL	156	30,997
West	W	146	14,760
NWCA aggregated wetland type			
Estuarine herbaceous	EH	258	20,186
Estuarine woody	EW	69	2015
Palustrine, riverine, or lacustrine-herbaceous	PRLH	302	55,038
Palustrine, riverine, or lacustrine-woody	PRLW	338	174,308

### Risk assessment variables

For our analyses, we assessed the relative and attributable risk of eight indicators of anthropogenic stress (hereon, called stressors) on wetland condition (Table 2). We chose six physical stressors (damming, ditching, filling/erosion, hardening, vegetation removal, and vegetation replacement) and two chemistry stressors (soil phosphorus and heavy metals) that represent a wide range of human disturbance activities. The six physical stressors are based on indices that consolidated all of the stressor indicators observed during field sampling into a manageable number of site-level groupings for risk analysis (Lomnicky et al. 2019). The two soil stressors were based on measured soil chemical concentrations (Nahlik et al. 2019).

**Table 2:** List of stressor categories, their description, field indicators, and the threshold values for defining high stressor levels using the anthropogenic stress index (ASI) for the stressor category

Stressor categories	Description	Field indicators*	High stressor-level threshold
Vegetation removal	Any field observation related to loss, removal, or damage of wetland vegetation	Gravel pit, wells, forest cut, highly grazed, recently burned, herbicide use, mowing/shrub cutting	Buffer-ASI ≥ 0.1
Vegetation replacement	Any field observation of altered vegetation within the site due to anthropogenic activities	Golf course, lawn/park, row crops, fallow field, nursery, orchard, tree plantation	Buffer-ASI ≥ 0.1
Damming	Any field observation related to impounding or impeding water flow from or within the site	Dike/dam/road/railroad bed, water level control structure, wall/riprap, berms	Buffer-ASI ≥ 0.1 OR AA-ASI ≥ 1.0
Ditching	Any field observation related to draining water	Ditches, channelization, inlets/outlets, point source/pipe, culverts	Buffer-ASI ≥ 0.1 OR AA-ASI ≥ 1.0
Hardening	Any field observation related to soil compaction, including activities and infrastructure that primarily result in soil hardening	Roads, parking lot/pavement, trails, soil compaction, dairy, residential, impervious surface, animal trampling	Buffer-ASI ≥ 0.1 OR AA-ASI ≥ 1.0
Filling/erosion	Any field observation related to soil erosion or deposition	Excavation/dredging, fill/spoil banks, freshly deposited sediment, soil erosion, irrigation, landfill, dumping	Buffer-ASI ≥ 0.1 OR AA-ASI ≥ 1.0
Heavy metals	Measured soil heavy metal (Ag, Cd, Co, Cr, Cu, Ni, Pb, Sb, Sn, V, W, Zn) concentrations	Uppermost soil layer with soil chemistry	Three or more metals > background concentrations (see, Nahlik et al. 2019)
Soil phosphorus	Measured soil phosphorus concentration by trace element protocol	Uppermost soil layer with soil chemistry	Set at the 95th percentile of the subpopulation reference distribution (USEPA 2016a)
			EH and EW = 969, Inland CPL = 1180, EMU = 1280, IPL = 1810, and W = 2090 mg P/kg

*Example human activity checklist items observed in the field for physical stressors, or location of soil samples for chemistry stressors

We used vascular vegetation as our measure of wetland condition as that was the primary condition variable collected in the NWCA. Thus, our risk assessments relate only to effects on plant community composition and not necessarily on overall wetland condition. Vascular plant species represent diverse adaptations, ecological tolerances, and life history strategies, and they integrate environmental factors, species interactions, and disturbance. Many disturbances are reflected in shifts in the presence or abundance of particular plant species, plant functional or trait groups, plant assemblages, or vegetation structural elements making vegetation a powerful indicator of wetland condition (Johnston et al. 2009, Mack and Kentula 2010, Magee et al. 2019). In the NWCA, vegetation condition was assessed using a vegetation multimetric index or VMMI (Magee et al. 2019).

### Stressor field and laboratory methods and index calculations

**Fig. 2:**
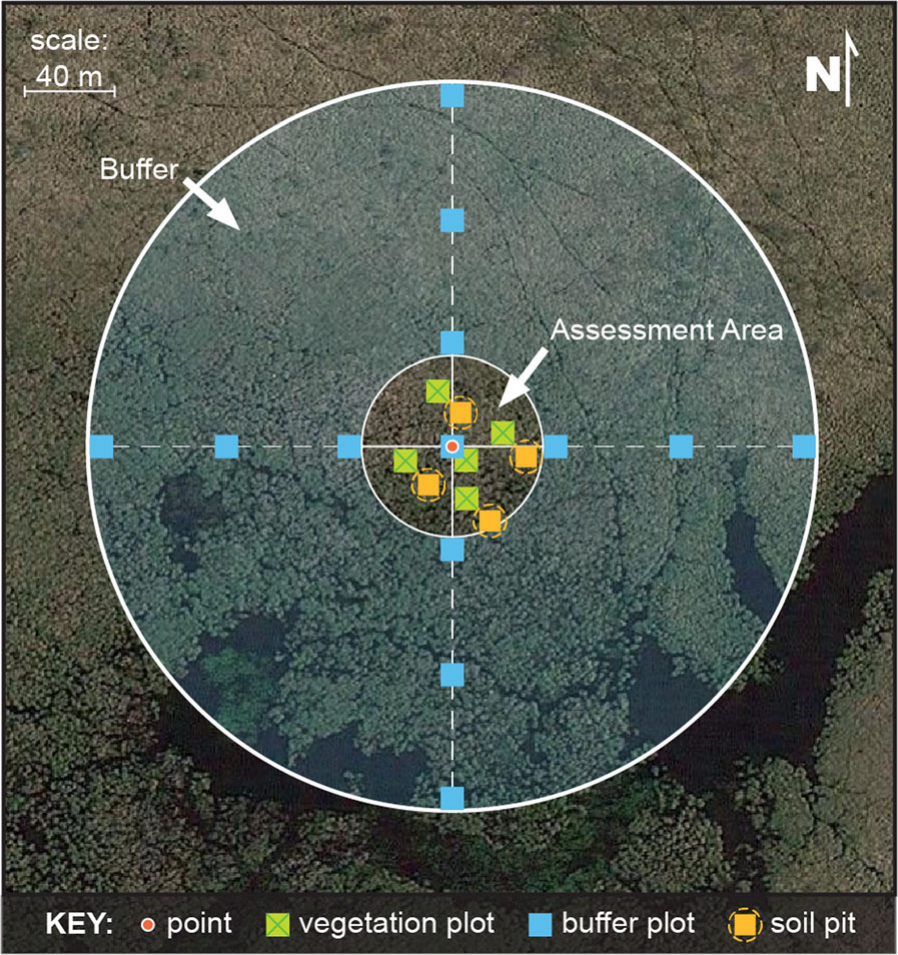
NWCA field sampling layout with the random center point (red dot), a central 40 m radius assessment area (AA), and additional 100-m radius buffer area. Sampling for vegetation, soils, and the hydrologic alteration checklist were conducted within the AA. Human activities in the buffer were tallied at the 13 10 × 10 m square buffer plots indicated by blue squares

Field and laboratory methods for the NWCA are described in detail by USEPA (2011a, 2011b). Wetland sites were sampled in 2011 during an index period ranging from April to September depending on the growing season of the state in which the site was located. Sample collection focused on a 0.5-ha assessment area (AA) defined around each selected sample point (Fig. 2). The AA was generally circular with a 40-m radius, but for very small or narrow wetlands, the AA shape was adjusted to a polygon or irregular shape to fit within the constraints of wetland boundaries. Within the AA, field crews: sampled soil (Nahlik et al. 2019) and vegetation (Magee et al. 2019), and completed checklists for presence of hydrologic alterations and other human activities (Lomnicky et al. 2019).

Four soil pit locations were systematically located in the AA (Fig. 2) and excavated to a depth of 60 cm. One soil pit was selected as representative of soil in the AA and expanded to a depth of 120 cm. At the representative pit, soil samples were collected for each soil layer more than 8-cm thick and sent to the lab for extensive chemical analysis (USEPA 2011a, 2011b). Due to difficulties in obtaining samples, 9.6% of the sites were missing soils data. Two soil chemical indicators of stress were developed—a heavy metal index (HMI; Nahlik et al. 2019) and soil phosphorus (soil P) concentrations (USEPA 2016a). Heavy metal concentrations are excellent indicators of stress: as heavy metals often have specific background ranges above which anthropogenic impacts are indicated. Soil P can be an important indicator of anthropogenic impacts (especially agricultural and residential stresses that result in eutrophication), but concentrations can be highly influenced by soil type, wetland type, region, and other factors. We only used data from the uppermost soil layer collected and analyzed from each site. Almost all (97%) sites from which soils were collected had chemistry data from a layer that began within 10 cm of the surface. In the laboratory, heavy metals and phosphorous were measured after nitric and hydrochloric acid extraction using an inductively coupled plasma mass spectrometer (USEPA 2011b). Twelve heavy metals (Table 2) were used to develop the HMI and it was scored as the sum of the number of the 12 metals present at any given site with concentrations above natural background levels based on published values, primarily from Alloway (2013) and reported in detail in Nahlik et al. (2019).

A checklist of hydrologic alterations observed anywhere within each AA was completed to create AA-based indices that were simply the integer number of observed alterations (Lomnicky et al. 2019). In addition, a checklist (hereafter, the buffer checklist data) of a wide variety of other descriptors of human activity was completed at 13 10 × 10 m plots, 1 located at the AA center, and 12 systematically arranged in the buffer surrounding the AA (Lomnicky et al. 2019). The 12 plots in the buffer were laid out in the 4 cardinal directions (3 in each direction): the first plot at the edge of the assessment area (40 m from the AA center), the second plot at the farthest extent of the study buffer (usually 140 m from the AA center), and the third plot midway between the other 2 (Fig. 2). The buffer checklist data and the AA hydrologic alteration checklist data were categorized into six physical anthropogenic stress indices (ASI): ditching, damming, filling/erosion, hardening, vegetation removal, and vegetation replacement (Table 2). A buffer-ASI was calculated for each of the six physical stressors based on the proximity-weighted average of the number of human activities for that category observed in each plot as described in Lomnicky et al. (2019). Disturbances in the AA plot and the inner ring of plots had a proximity weight of 1, the middle ring plots had a weight of 0.44, and the outer ring of plots had a weight of 0.23. The index value was calculated as the sum of the number of specific disturbance tallies in each plot times the plot proximity weight, summed across all plots at the site, and then divided by the total number of plots (13 for almost all sites). Thus, if there was one stressor activity observed at each of the 13 plots distributed within the wetland and its surrounding buffer area, the ASI index score would be 0.59. The maximum value observed at any site in the NWCA for any physical ASI was 2.2 but it was rare for a site to have values greater than 1.

### Vegetation field methods and index calculation

Vegetation sampling methods are described in detail elsewhere (USEPA 2011a; Magee et al. 2019) and summarized here. Five 100-m^2^ vegetation plots were systematically placed in the AA (Fig. 2) according to predetermined rules based on the shape of the AA. All vascular plants in each plot were identified to the lowest taxonomic level possible, typically to species. Taxa not readily identified in the field were collected and identified in the lab by regionally expert botanists. Percent cover for each species was estimated as a direct percentage (0–100%) of the 100-m^2^ area of each vegetation plot. Species trait information, including state-level coefficients of conservatism (*C* values) and state-level native status, was gathered from literature or database sources, or in some cases developed, for each taxon-state pair observed in the NWCA (USEPA 2016a; Magee et al. 2019).

The field data and species trait information were used to calculate numerous candidate metrics of vegetation condition (*n* = 405), which were screened based on range, redundancy, repeatability, and responsiveness, for potential inclusion in a VMMI that would serve as the principal indicator of biological condition for the NWCA (USEPA 2016b). VMMI development, calculation, and use are detailed in Magee et al. (2019). In brief, 35 of the candidate metrics effectively distinguished least-disturbed (reference) from most-disturbed sites and were considered as potential VMMI components. A permutation approach was used to calculate thousands of randomly constructed candidate national-scale VMMIs based on combinations of 4, 6, 8, and 10 metrics. The candidate VMMIs were quantitatively evaluated based on limited redundancy among constituent metrics, sensitivity, repeatability, and precision. The final VMMI is composed of four broadly applicable metrics as detailed in Magee et al. (2019). The first two metrics were the floristic quality assessment index (which is based on species *C* values), and the relative importance of native plants (calculated as the sum of the relative cover and frequency of native plant species). The final two metrics were the number of plant species tolerant to disturbance (those with *C* values ≤ 4) and the relative cover of native monocots. Each metric was scored from 0 to 10, the four scores were summed, and multiplied by 100/40 so that the final VMMI ranged from 0 to 100 with higher values reflecting better condition (Magee et al. 2019).

### Relative and attributable risk calculation

Relative and attributable risk are calculated using class data, specifically a 2 × 2 contingency table of condition class versus stressor-level class. We used the stressor-level classes (Table 2) and vegetation condition classes developed for the NWCA (USEPA 2016a) for our risk analysis. In the NWCA, wetland ecological condition is defined at each site as good, fair, or poor based on the site VMMI value. To account for natural variation in the VMMI across the conterminous US, different VMMI value thresholds for delineating good, fair, and poor condition were defined for each of the ten ecoregion-by-wetland-type reporting groups as detailed in Magee et al. (2019). The reporting groups were derived by crossing the wetland types and ecoregions depicted in Fig. 1 as described in Herlihy et al. (2019). The exact condition thresholds were calculated from the distribution percentiles for VMMI values at reference (least-disturbed) sites in each reporting group. To obtain two condition categories for use in the contingency table, we compared a not poor condition class (i.e., the combination of good and fair condition) to the poor condition class. We combined good and fair condition, because the objective of reporting relative risk in the NWCA is to indicate which stressors policy makers and managers may want to prioritize for management efforts aimed at decreasing wetland area that is poor condition.

In the NWCA, sites were categorized into low, moderate, and high stressor-level classes. A reference site distribution percentile approach was also used to set specific soil P class thresholds for each reporting group using the same methodology as that used for the vegetation condition classes (USEPA 2016a). Uniform thresholds across the US were used to define stressor-level classes for heavy metals and physical stressors (Table 2). The low stressor-level class was defined as having an index value of zero. For the physical stressors, the high stressor-level threshold was assigned using best professional judgment, and the stressor-level threshold differs between the buffer-based and AA-based ASIs (USEPA 2016a). To be considered in the high stressor-level class, a site had to exceed either a value ≥ 0.1 for the buffer-ASI or a value ≥ 1.0 for the AA-ASI (Table 2). A buffer-ASI of ≥ 0.1 means that, for example, at least two disturbances from the checklist were observed in or within closest proximity to the AA, or at least six disturbances were observed in the farthest proximity to the AA. On the other hand, AA-ASI are integers, and a value of ≥ 1.0 represents one or more observations of hydrologic alteration anywhere within the AA. Sites that were not low or high stressor-levels were considered moderate. As was done for vegetation condition classes, two classes were defined as not high stressor-level (i.e., a combination of low and moderate) and high stressor-level.

To calculate relative risk, a 2 × 2 matrix or contingency table of stressor-level class versus condition class was created. The population weights were used to calculate the proportion of the wetland population (by area) that is in each of the cells of the matrix (e.g., poor wetland condition and high stressor level). The relative risk ratio (RR) is calculated as the ratio of two proportions,

$$ \mathrm{RR}=\frac{\Pr\ \left(\mathrm{poor}\kern0.5em \mathrm{condition},\kern0.5em \mathrm{given}\kern0.5em \mathrm{high}\kern0.5em \mathrm{stressor}\right)}{\Pr\ \left(\mathrm{poor}\ \mathrm{condition},\kern0.5em \mathrm{given}\kern0.5em \mathrm{not}\ \mathrm{high}\kern0.5em \mathrm{stressor}\right)} $$

where Pr is the proportion of wetland area. A relative risk value of 1.0 indicates that there is no association between the stressor and the biological indicator, while values greater than 1.0 suggest greater relative risk. For example, if 30% of the population is in poor condition but it is equally divided among sites with high and not high stressor levels (15% in each), then the RR = 0.15/0.15 = 1, and there is no association between condition and the stressor. Conversely, if the 30% in poor condition was observed as 25% in sites with high stressor level and 5% in sites with not high stressor level, then the RR = 25/5 = 5.0. The higher the relative risk value for a given stressor, the greater the risk of poor wetland condition. A relative risk of 5 indicates that we are five times more likely to see a wetland in poor condition when the stressor is in the high category than when it is in the not high category. Statistical confidence intervals around each relative risk ratio were calculated as described by Van Sickle et al. (2006) to assess significant differences among stressors (Van Sickle and Paulsen 2008). When the lower 95% confidence interval for any given relative risk ratio falls below 1.0, we do not consider relative risk to be statistically significant. These confidence intervals can also be used to assess significant differences among stressors (Van Sickle and Paulsen 2008).

Attributable risk combines estimated stressor extent with relative risk into a single index and was calculated using the following formula (Van Sickle and Paulsen 2008):

$$ \mathrm{AR}=\frac{\Pr\ \left(\mathrm{high}\ \mathrm{stressor}\ \mathrm{levels}\right)\times \left(\mathrm{RR}-1\right)}{1+\Pr\ \left(\mathrm{high}\ \mathrm{stressor}\ \mathrm{levels}\right)\times \left(\mathrm{RR}-1\right)} $$

where AR is attributable risk, RR is relative risk and Pr is proportion of wetland area in the population. The same condition class and stressor-level classes were used for calculating attributable risk as relative risk (i.e., not poor and not high was compared to poor and high condition classes and stressor levels, respectively). Confidence bounds on attributable risk were calculated using the method of Van Sickle and Paulsen (2008).

Relative and attributable risk are relative measures and depend on the population being analyzed. Following the health analogy, the relative risk of a stressor is usually different for the entire population than it is for just young males. We calculated national relative and attributable risk estimates for the conterminous US using NWCA data from all the sampled probability sites (*n* = 967). We also wanted to examine relative and attributable risks at smaller scales. Thus, we also calculated relative and attributable risk for both the four ecoregions and four wetland types in Table 1 using just those sites that fell within each subpopulation.

## Results and discussion

### National Results

Based on the survey design and the field visits made in the NWCA, the 967 probability sites are a spatially balanced representative sample of the 251,546 km^2^ of sampleable wetland area in the target population in the conterminous US (see, Olsen et al. (2019) for details). Half of this wetland area is located in the Coastal Plain (CPL), 32% is in the Eastern Mountains and Upper Midwest (EMU), and only 18% in the Interior Plains (IPL) and West (W) ecoregions combined (Table 1). Across wetland types, 70% of the area is comprised of woody versus 30% herbaceous systems. The vast majority (69%) of the estimated wetland area in the NWCA target population is in the palustrine, riverine, or lacustrine–woody (PRLW) wetland type. Most of the wetland area is represented by inland wetland types (PRLW and palustrine, riverine, or lacustrine–herbaceous (PRLH)), with only 8.8% of the area in estuarine (estuarine–herbaceous (EH) and estuarine–woody (EW)) types (Table 1). Thus, even in the CPL ecoregion, most of the wetland area is freshwater dominated. All of the following risk analyses are relative to these areal population estimates of the NWCA target population.

In terms of wetland ecological condition at the national scale, as indexed by the VMMI, 48.3% of the wetland area was in good condition, 19.6% was in fair condition, and 32.1% was in poor condition (Table 3). Among ecoregions, the West had the highest percentage of wetland area in poor condition (60.7%) and the lowest percentage in good condition (21.5%). The percentage of area in good condition was fairly similar among the other three ecoregions. Estuarine wetland types had a higher percentage of wetland area in good condition and a lower percentage in poor condition than inland types (Table 3). There were only minor differences in vegetation condition between woody and herbaceous wetland types within estuarine or inland type.

**Table 3:** NWCA population estimates of ecological condition expressed as percent of wetland area in good, fair, or poor condition based on the vegetation multimetric index (VMMI), nationally and by aggregated ecoregion and wetland type

Full name	Code	% Good	% Fair	% Poor
National	ALL	48.3	19.6	32.1
NWCA aggregated ecoregion				
Coastal Plain	CPL	50.1	21.4	28.5
Eastern Mountains and Upper Midwest	EMU	52.0	10.6	37.4
Interior Plains	IPL	44.1	36.5	19.5
West	W	21.5	17.8	60.7
NWCA aggregated wetland type				
Estuarine herbaceous	EH	57.8	16.6	25.7
Estuarine woody	EW	58.5	19.6	21.9
Palustrine, riverine, or lacustrine-herbaceous	PRLH	51.0	16.0	33.0
Palustrine, riverine, or lacustrine-woody	PRLW	46.2	21.1	32.7

The total estimated wetland area for each subpopulation is given in Table 1

**Fig. 3:**
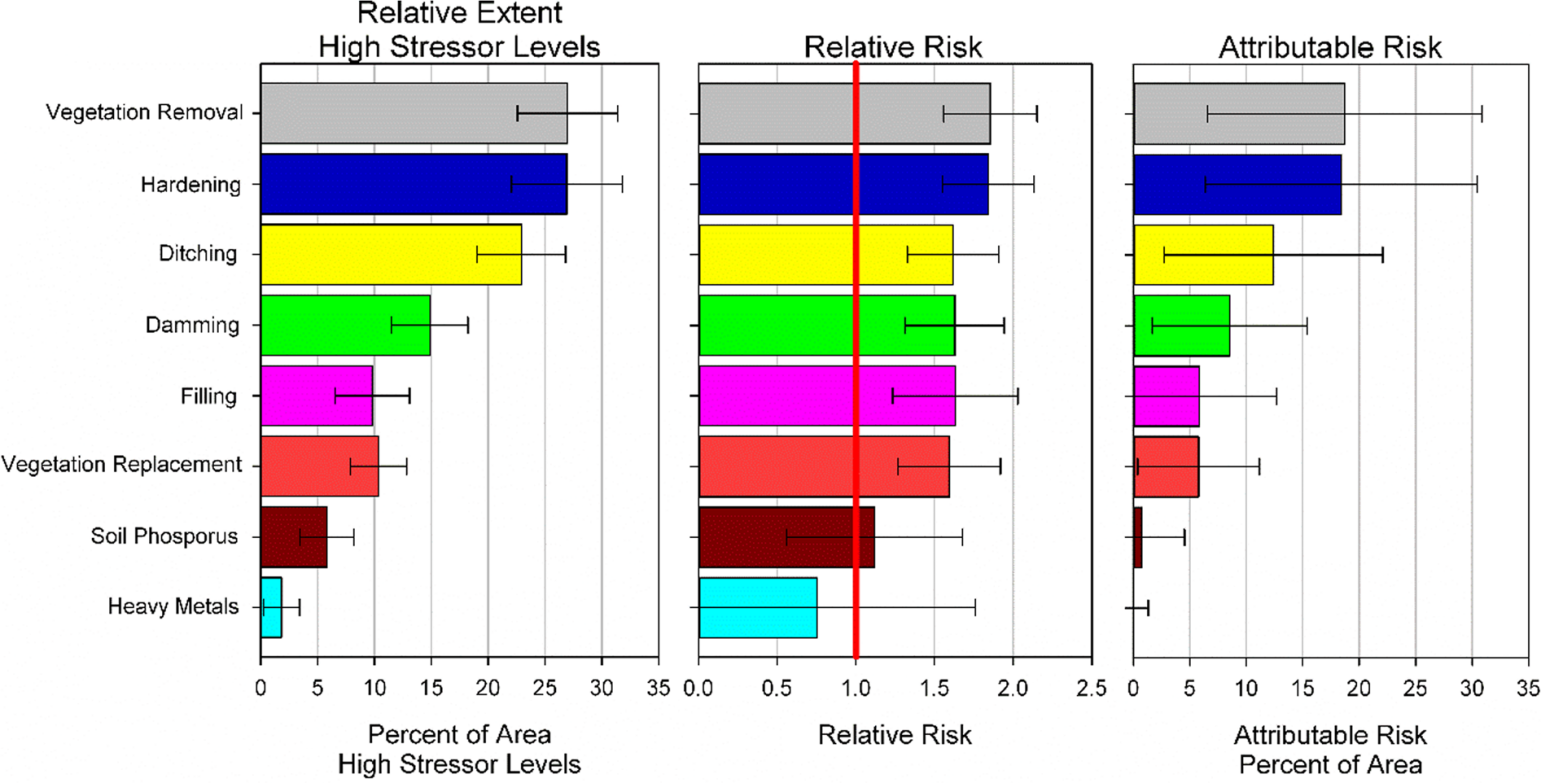
National-level estimates for relative extent of stressor indicators when stressor level is high, relative risk associated with each stressor indictor, and attributable risk for each stressor indicator relative to wetland vegetation condition

Nationally, vegetation removal and hardening were the stressors that most commonly had the greatest extent of wetland area with high stressor levels (relative extent, Fig. 3). Both of these stressors were categorized in the high stressor level in an estimated 27% of the NWCA wetland area. Ditching and damming had high stressor level in 15–25% of the area nationally. In contrast, high stressor levels from soil stressors appeared relatively rare, with only 5% of the area having soil P and 2% heavy metal falling into the high stressor level.

Relative risk nationally was similar among the six physical stressors (Fig. 3). All six had significant relative risk with values between 1.6 and 1.8 indicating the likelihood of having poor vegetation condition was just under twice as likely in sites with high physical stress than low stress. The relative risk of soil P and heavy metals was not significant (the lower 95% confidence interval was < 1).

Similar relative risk values among the physical stressors meant that attributable risk was driven primarily by the relative extent of stressors with high stressor levels. Attributable risk was highest for vegetation removal and hardening at 19% (Fig. 3), indicating that 19% of the wetland area in poor condition might be improved to non-poor (i.e., good or fair condition) if the high stressor levels were eliminated. attributable risk of the other physical stressors ranged from 5 to 12%. Soil P and heavy metals had virtually 0% attributable risk.

The calculation of attributable risk does make three major assumptions about (1) causality (the stressor causes an increased probability of poor condition); (2) reversibility (if the stressor is eliminated, causal effects will also be eliminated); and (3) independence (stressors are independent of each other). Of these, independence is probably the most problematic. The presence of human activity tends to generate multiple stressors rather than just one single stressor. These major assumptions must be considered when applying the attributable risk results to management decisions. Nevertheless, attributable risk provides much needed insight into how one might prioritize management for the improvement of our Nation’s aquatic ecosystems—wetlands, in the case of the NWCA. While the results of attributable risk estimates are presented as absolutes (i.e., the percent area in poor condition that could be reduced if the effects of a particular stressor were eliminated), these estimates probably better serve as general guidance as to what stressors are affecting condition and to what degree (relative to the other stressors evaluated).

There are some similarities between our relative risk analyses and regression analyses of stressor versus VMMI scores. They both use the exact same underlying data to examine the associations among variables. The big difference is that relative risk breaks the continuous stressor and VMMI data into classes before analysis. As such, it is far easier for lay audiences to understand relative risk as opposed to *r*^2^ values from regressions. Relative risk, in our analysis, requires breaking the continuous variables into discrete groups and the results are dependent on the thresholds used to define the groups. Herlihy et al. (2019b) did a regression analysis of both field and landscape stressors as independent variables versus VMMI score as the dependent variable using the same NWCA data and subpopulations used here for relative risk analysis. There were no observed regression models with *r*^2^ > 0.4. The best multiple regression model nationally, had an *r*^2^ = 0.251 and included damming, ditching, vegetation removal, and percent agriculture and development in a 1-km radius buffer around the site as independent variables. The strongest individual ecoregion or wetland-type regression model was for the EMU (*r*^2^ = 0.374) and included vegetation replacement, percent agriculture, and percent development terms.

Risk estimates for the NWCA varied from those observed for other NARS in the US. For example, in the National Rivers and Streams Assessment (NRSA), water column nutrients (total phosphorus and total nitrogen) had the largest attributable risks to aquatic invertebrates of 30 and 26%, respectively, while attributable risk for the four physical habitat indicators ranged from 5 to 16% (USEPA 2016c). The relative risk values for NRSA were more similar among the nutrient and physical habitat stressors, ranging from 1.3 to 1.9. For the National Lakes Assessment (NLA) similar risk data were generated (USEPA 2016d). Attributable risk for total phosphorus and total nitrogen on lake macroinvertebrates were 35 and 16%, respectively. The attributable risk for the physical indicators ranged from 9 to 12%. Relative risk, on the other hand, was 2.2 for TP and 1.5 for TN, and ranged from 1.0 to 1.6 for the physical habitat indicators in lakes. It should be noted, however, that for these NARS lakes and stream risk estimates, ecological condition was assessed using aquatic invertebrates as opposed to the use of vegetation in the NWCA. Risk estimates will vary depending on the biological assemblage used to assess condition. For example, NRSA risk estimates for aquatic invertebrates differ from those using fish assemblages to define condition (USEPA 2016c). Thus, any direct comparison of wetland to lake and stream results should be interpreted with an appreciation of the very different assemblages involved.

### Ecoregion subpopulation results

**Fig. 4:**
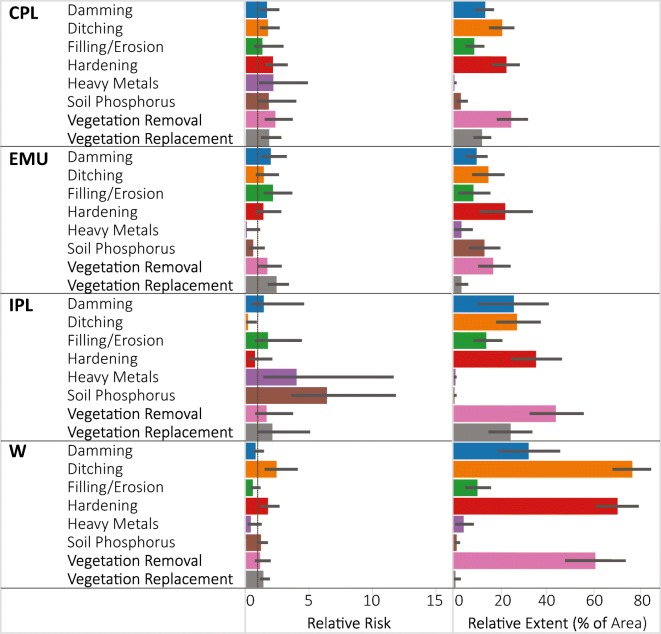
NWCA estimates of stressor relative risk and relative extent (% of wetland area with high stressor levels) presented by NWCA aggregated ecoregion. Error bars are 95% confidence intervals. Ecoregion codes are given in Table 1

The relative extent of high stressor levels varied widely among ecoregions (Fig. 4). For example, the high stressor level for ditching was observed in 70% of the wetland area in the W, but in only 10% of the EMU. Vegetation replacement had a high stressor level in 24% of the IPL wetland area, but was rare (< 4%) in the EMU and W. None of the stressors had a particularly large relative extent in the EMU (all < 20%) or CPL (all < 25%). The majority of the wetland area in the W had high stressor levels for ditching, hardening, and vegetation removal. As discussed in more detail in Nahlik et al. 2019), high levels for heavy metal stress were rare in all ecoregions.

Only two of the evaluated stressors had significant relative risk to wetland condition in the IPL, soil P at 6.5 and heavy metals at 4.0; however, these values reflected the greatest relative risk observed for any ecoregion (Fig. 4). While these soil stressors may currently be rare (i.e., small relative extent with high stressor level) in the IPL, when they do occur, the risk of having poor vegetation condition is high. It should be noted, however, that due to the small number of sites with high soil P and heavy metal stressor levels in the IPL, the uncertainty in the exact relative risk estimate is very high (large error bars in Fig. 4). In the CPL, all the stressors except filling/erosion and soil P had a significant relative risk with similar values (1.8 to 2). In the EMU, damming, filling/erosion and vegetation replacement both posed significant relative risk (values ~ 2) to wetland condition, whereas in the W, significant relative risk values of 1.5 to 2 were observed for ditching, hardening, and vegetation replacement.

The size of the 95% confidence bounds for relative extent, relative risk, and attributable risk are largely driven by overall sample size and the distribution of sampled sites and their sample weights among the cells of the 2 × 2 contingency matrix. Thus, confidence bounds are narrower nationally than for any of the individual ecoregions or wetland types. Also, subpopulations with larger sample sizes like the CPL generally have narrower confidence bounds than those with smaller sample sizes (see, Table 1 for sample sizes).

**Fig. 5:**
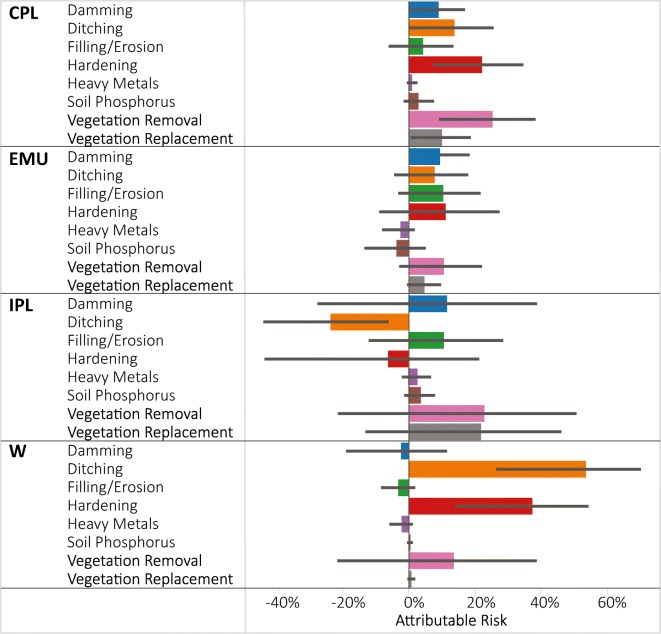
Attributable risk estimates of % wetland area in poor vegetation condition that could be improved to non-poor if high stressor levels were removed presented by NWCA aggregated ecoregion**.** Error bars are 95% confidence intervals. Ecoregion codes are given in Table 1

Across ecoregions, the highest attributable risks were observed in the W for ditching (58%) and hardening (39%) indicating the biggest potential for ameliorating poor vegetation condition occurs in the W by reducing high levels of those two stressors (Fig. 5). Attributable risk was also above 20% for hardening and vegetation removal in the CPL, and for vegetation removal and vegetation replacement in the IPL. Attributable risk was < 10% for all stressors in the EMU. Some of the stressors had negative attributable risk which occurs when relative risk is < 1. When relative risk is < 1, a positive association exists between the stressor and condition such that poor condition is less likely to be observed when stressor levels are high. If the 95% confidence bound around relative risk encompasses 1, it indicates that both relative and attributable risk are not significant in either direction. With the exception of ditching in the IPL, in all of the instances where the relative risk was < 1 (Fig. 4), the upper 95% confidence bound exceeded 1; thus, we do not consider them to be significantly < 1. From our data, it’s not possible to determine why ditching in the IPL was significantly related to not poor vegetation condition.

### Wetland-type subpopulation results

**Fig. 6:**
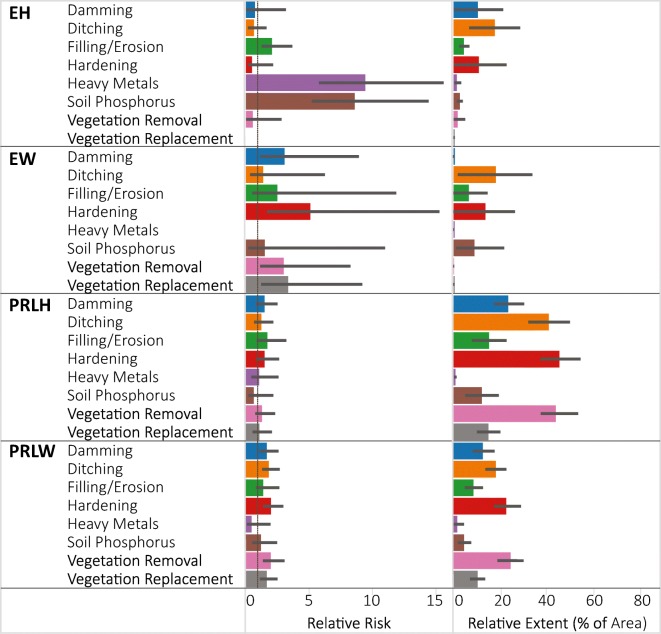
NWCA estimates of stressor relative risk and relative extent (% of wetland area with high stressor levels) presented by NWCA aggregated wetland type. Relative risk for vegetation replacement in EH and heavy metals in EW were indeterminate due to the absence of those stressors in those subpopulations. Error bars are 95% confidence intervals. Wetland type codes are given in Table 1

The areal extent of high stressor levels in the estuarine (EH and EW) wetland types tended to be much lower than that observed in the inland (PRLH and PRLW) wetland types (Fig. 6). In estuarine wetlands, ditching was the most prevalent stressor in both EH (20% of area) and EW (21% of area). Most of the other evaluated stressors occurred at high stressor levels in < 10% of the wetland area in either estuarine type. In the PRLH wetland type, high ditching, hardening, and vegetation removal stressor levels were present in over 40% of the wetland area. These were also the most prevalent stressors by areal extent in the PRLW as well, albeit at lower percentages (20–25%). High levels of soil heavy metals were rare (< 5% of area) in all wetland types.

In the EH, the relative risk for poor condition with high stressor levels for both heavy metals and soil P was > 8; the greatest relative risk values observed in our analyses (Fig. 6). These two stressors were rare in the EH with a low relative extent of high stressor levels, but in places where high levels occurred, there was a high risk of poor vegetation condition. As with the soil stressors in the IPL ecoregion (Fig. 4), there is a high level of uncertainty in the exact relative risk value for soil stressors in the EH due their rarity (low sample size of high stressor-level sites). Relative risk was also high (> 2) and significant for filling/erosion in the EH, and for damming, hardening, vegetation removal, and vegetation replacement in the EW. Hardening and vegetation removal also had significant relative risk (> 2) in the PRLW. In contrast, none of the observed stressors showed significant relative risk in the PRLH, despite most having relatively large extent categorized in the high stressor level (Fig. 6). The lack of significant relative risk results in the PRLH and the lower values in PRLW relative to the estuarine types is likely related to the wide geographic extent and the diverse set of wetlands that were combined to form the aggregated palustrine, riverine, or lacustrine (PRL) wetland type. In other work, Herlihy et al. (2019b) found a wide variety of stressor-condition responses within different NWCA wetland types and ecoregions. Thus, in larger, more heterogeneous subpopulations, the association of a single stressor-condition response may be blurred by this variability resulting in somewhat lower relative risk values. This may also explain why, at the national scale, all the stressors had relative risk values less than 2.

**Fig. 7:**
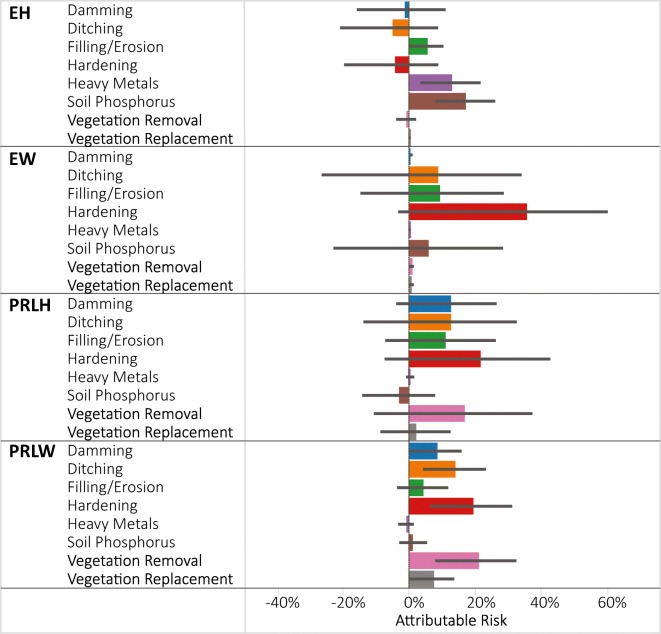
Attributable risk estimates of % wetland area in poor vegetation condition that could be improved to non-poor if high stressor levels were removed presented by NWCA aggregated wetland type. Attributable risk for vegetation replacement in EH and heavy metals in EW were indeterminate due to the absence of those stressors in those subpopulations. Error bars are 95% confidence intervals. Wetland type codes are given in Table 1

The largest attributable risk observed by wetland type was 37% for hardening in the EW (Fig. 7), suggesting that 37% of the estuarine woody wetland area that was in poor condition might be improved if high hardening stressor levels could be reversed. At minimum, since this is a highly ranked indicator of stress, and once hardening effects are in place, they may be difficult to reverse, management actions might be prioritized to attempt to prevent or decrease the occurrence of hardening disturbances. Other stressors with attributable risk > 15% (and had significant relative risk > 1) included soil P in the EH, and hardening and vegetation removal in the PRLW. Attributable risk for heavy metals and soil P in the EH were only 12 and 16%, respectively, even though their relative risks were > 8 because their extent in the high stressor-level category was small (< 4%, Fig. 6). Negative attributable risk was only observed for a few stressors (Fig. 7) among the wetland type groups and all were non-significant.

### Synthesis and conclusions

**Fig. 8:**
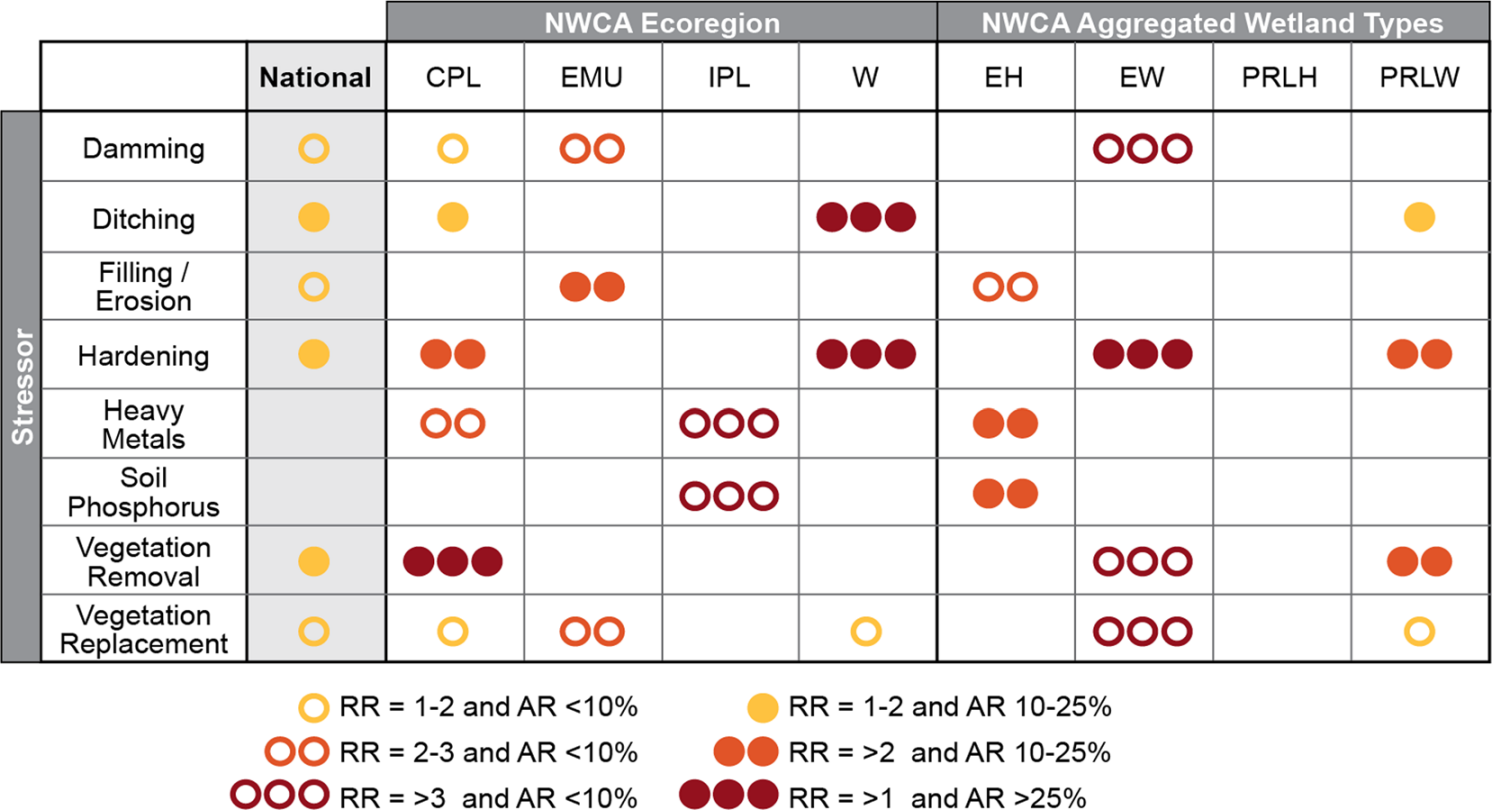
**S**ummary of significant relative risk (RR) and attributable risk (AR) levels nationally and by NWCA aggregated ecoregion and wetland type. Ecoregion and wetland type codes are given in Table 1

We synthesized the results of all the risk analyses into one figure (Fig. 8) to allow comparison of the evaluated stressor indicators nationally, and across all ecoregion and wetland type subpopulations. The figure indicates that six of the stressor indicators were significant at the national scale, and that no single stressor is predominant across all ecoregions or wetland types. All of the stressors were associated with poor vegetation condition in one or more of the subpopulations. Thus, the risk analyses completed in this study were strongly dependent on the scale being assessed, with the risk being specific to the population being evaluated. For example, a stressor, such as heavy metals may have no observed relative risk nationally or within most subpopulations, but have high relative risk in a specific subpopulation like the IPL. Among all evaluated stressors, hardening had the highest attributable and relative risks in the greatest number of subpopulations. Attributable risks above 25% were observed for vegetation removal in the CPL, hardening and ditching in the W, and hardening in the EW. Relative risks above 3 were noted for soil heavy metals and soil P in the IPL, and vegetation removal, vegetation replacement, and damming in the EW.

Relative risk gives one an idea of how severe of a biological impact is likely to occur when high levels of stress occur. This is most useful to site managers and in deciding on actions site-by-site. Ranking stressors by relative risk allows a way to set priorities at that site and helps weigh options between addressing one stressor over another. Relative risk by itself, however, may not be an effective tool when looking to rank stressors across broad regions or wetland types. Although individual stressors might have high relative risk, if the stressor does not occur in high levels at very many locations, then maybe it should not be a regional or national priority as addressing that particular stressor will not benefit much of the resource. Nevertheless, a stressor that poses extremely high relative risk, but currently influences only a limited area, may warrant monitoring for increases in extent across a region.

Attributable risk combines the “effect size” (relative risk) with how widespread (relative extent) the stressor is, and, when ranked against other stressors, provides a sense of how much overall improvement in wetland conditions would occur by tackling each stressor compared with the others. Figures 5 and 7 illustrate which stressors are associated with the greatest attributable risk by region and wetland type, and thus the potential benefit for each subpopulation that might be possible if high stressor levels could be removed. From a national policy perspective, one could just look at the ranking of stressors using an attributable risk figure (e.g., Fig. 3). This figure clearly shows that the greatest overall benefit to wetlands nationally would occur if vegetation removal and hardening were addressed. Figures 5 and 7 demonstrate that the largest attributable risk, and thus expected benefit from addressing particular stressors would vary significantly by region and wetland type.

Relative risk and attributable risk were added to the data analyses tools in NWCA and NARS to improve the ability of the survey results to assist managers and policy makers in setting priorities based on conditions on the ground. Clearly, other factors (e.g., economics, ecosystem services, stakeholder needs) will come into play when making management decisions, but the risk measures offer unique information that may help in prioritizing management or monitoring actions. For wetland managers focused primarily on individual wetland problems, examining the relative risk is probably the more useful statistic (as opposed to attributable risk). Relative risk relates the likelihood that a specific stressor could result in poor condition. Such information would aid in decision-making about which stressors to reduce or eliminate (or prevent from happening) at the site. From our view, analyses of relative and attributable risk provide useful information to both individual site managers and regional-national policy makers.

## References

[CR1] Alloway BJ (2013). Heavy metals in soils: trace metals and metalloids in soils and their bioavailability.

[CR2] American Cancer Society (2017). Colorectal cancer facts & figures 2017-2019.

[CR3] Butterworth AS, Higgins JP, Pharoah P (2006). Relative and absolute risk of colorectal cancer for individuals with a family history: a meta-analysis. European Journal of Cancer.

[CR4] Herlihy, A. T., Kentula, M. E., Magee, T. K., Lomnicky, G. A., Nahlik, A. M., Serenbetz, G. (2019a). Striving for consistency in the National Wetland Condition Assessment: developing a reference condition approach for assessing wetlands at a continental scale. *Environmental Monitoring and Assessment*. 10.1007/s10661-019-7325-3. 10.1007/s10661-019-7325-3 PMC6586693 31222681

[CR5] Herlihy, A. T., Sifneos, J. C., Lomnicky, G. A., Nahlik, A. M., Kentula, M. E., Magee, T. K., Weber, M. H., & Trebitz, A. S. (2019b). The response of wetland quality indicators to human disturbance across the United States. *Environmental Monitoring and Assessment*. 10.1007/s10661-019-7323-5. 10.1007/s10661-019-7323-5 PMC6586913 31222417

[CR6] Johnston CA, Zedler JB, Tulbure MG, Frieswyk CB, Bedford BL, Vaccaro L (2009). A unifying approach for evaluating the condition of wetland plant communities and identifying related stressors. Ecol Appl.

[CR7] Kaufmann PR, Herlihy AT, Mitch ME, Messer JJ, Overton WS (1991). Chemical characteristics of streams in the Eastern United States: I. Synoptic survey design, acid-base status and regional chemical patterns. Water Resources Research.

[CR8] Larsen DP, Thornton KW, Urquhart NS, Paulsen SG (1994). The role of sample surveys for monitoring the condition of the nation’s lakes. Environmental Monitoring and Assessment.

[CR9] Landers DH, Overton WS, Linthurst RA, Brakke DF (1988). Eastern Lake Survey: regional estimates of lake chemistry. Environmental Science & Technology.

[CR10] Lomnicky, G.A., Herlihy, A. T., Kaufmann, P. R. (2019). Quantifying the extent of human disturbance activities and anthropogenic stressors in wetlands across the conterminous United States – results from the National Wetland Condition Assessment. *Environmental Monitoring and Assessment*. 10.1007/s10661-019-7314-6. 10.1007/s10661-019-7314-6 PMC6586716 31222443

[CR11] Mack JJ, Kentula ME (2010). Metric similarity in vegetation-based wetland assessment methods. EPA/600/R-10/140.

[CR12] Magee, T.K., Blocksom, K.A., & Fennessy, M.S. (2019). A national-scale vegetation multimetric index (VMMI) as an indicator of wetland condition across the conterminous United States. 10.1007/s10661-019-7324-4. 10.1007/s10661-019-7324-4 PMC6586711 31222469

[CR13] Nahlik, A. M., Blocksom, K. A., Herlihy, A. T., Kentula, M. E., Magee T.K., and Paulsen, S.G. (2019). Use of national-scale data to examine human-mediated additions of heavy metals to wetland soils of the United States. *Environmental Monitoring and Assessment*. 10.1007/s10661-019-7315-5. 10.1007/s10661-019-7315-5 PMC6586720 31222398

[CR14] Olsen, A.R., Kincaid, T.M., Kentula, M.E., & Weber, M.H. (2019). Survey design to assess condition of wetlands in the United States. 10.1007/s10661-019-7322-6. 10.1007/s10661-019-7322-6 PMC6586691 31222669

[CR15] Paulsen SG, Mayio A, Peck DV, Stoddard JL, Tarquinio E, Holdsworth S, Van Sickle J, Yuan LL, Hawkins CP, Herlihy AT, Kaufmann PR, Barbour MT, Larsen DP, Olsen AR (2008). Condition of stream ecosystems in the US: an overview of the first national assessment. Journal of the North American Benthological Society.

[CR16] Stevens DL, Olsen AR (1999). Spatially restricted surveys over time for aquatic resources. Journal of Agricultural, Biological, and Environmental Statistics.

[CR17] Stevens DL, Olsen AR (2004). Spatially-balanced sampling of natural resources. Journal of the American Statistical Association.

[CR18] USEPA (2011). National Wetland Condition Assessment: field operations manual. EPA/843/R10/001.

[CR19] USEPA (2011). National Wetland Condition Assessment: laboratory operations manual. EPA-843-R-10-002.

[CR20] USEPA (2016). National Wetland Condition Assessment: 2011 technical report. EPA-843-R-15-006.

[CR21] USEPA (2016). National Wetland Condition Assessment 2011: a collaborative survey of the nation’s wetlands. EPA-843-R-15-005.

[CR22] USEPA (2016). National Rivers and Streams Assessment 2008-2009: a collaborative survey*.* EPA-841-R-16-007.

[CR23] USEPA (2016). National Lakes Assessment 2012: a collaborative survey of lakes in the United States. EPA 841-R-16-113.

[CR24] Van Sickle J, Stoddard JL, Paulsen SG, Olsen AR (2006). Using relative risk to compare the effects of aquatic stressors at a regional scale. Environmental Management.

[CR25] Van Sickle J, Paulsen SG (2008). Assessing the attributable risks, relative risks, and regional extents of aquatic stressors. Journal of the North American Benthological Society.

[CR26] Van Sickle J (2013). Estimating the risks of multiple, covarying stressors in the National Lakes Assessment. Freshwater Science.

